# Experimental Characterization of a Silicon Nitride Asymmetric Loop-Terminated Mach-Zehnder Interferometer with a Refractive Index-Engineered Sensing Arm

**DOI:** 10.3390/nano15191532

**Published:** 2025-10-08

**Authors:** Muhammad A. Butt, Mateusz Słowikowski, Dagmara Drecka, Michał Jarosik, Ryszard Piramidowicz

**Affiliations:** 1 Institute of Microelectronics and Optoelectronics, Warsaw University of Technology, Koszykowa 75, 00-662 Warsaw, Poland; 2 The Centre for Advanced Materials and Technologies CEZAMAT, Warsaw University of Technology, Poleczki 19, 00-822 Warsaw, Poland

**Keywords:** loop-terminated Mach-Zehnder Interferometer, silicon nitride, refractive index sensor, subwavelength grating

## Abstract

We report the design, fabrication, and experimental characterization of an asymmetric loop-terminated Mach–Zehnder interferometer (a-LT-MZI) realized on a silicon nitride (SiN) platform for refractive index (RI) sensing. The LT-MZI architecture incorporates a Sagnac loop that enables bidirectional light propagation, effectively doubling the interaction length without enlarging the device footprint, enhancing sensitivity and improving stability against environmental noise. Subwavelength grating (SWG) waveguides were integrated into the sensing arm to further strengthen light-matter interaction. The fabricated devices exhibited stable and well-defined interference fringes, with uniform wavelength shifts that scaled linearly with changes in the surrounding refractive index. Standard a-LT-MZI structures (ΔL = 300 μm) achieved experimental sensitivities of 288.75–301.25 nm/RIU, while SWG-enhanced devices reached 496–518 nm/RIU, confirming the effectiveness of refractive index engineering. Comparative analysis against previously reported MZI-based sensors highlights the competitive performance of the proposed design. By combining the scalability and CMOS compatibility of silicon nitride with the sensitivity and robustness of the a-LT-MZI architecture, this device provides a compact and versatile platform for next-generation lab-on-chip photonic sensors. It holds strong potential for applications in biochemical diagnostics, medical testing, and environmental monitoring.

## 1. Introduction

The loop-terminated Mach-Zehnder interferometer (LT-MZI) is an advanced interferometric configuration that has recently emerged as a promising candidate for highly sensitive and compact optical sensing [1]. In contrast to conventional MZIs, where two independent arms recombine after accumulating a relative phase difference, the LT-MZI introduces a Sagnac loop at one end, enabling bidirectional light propagation [2]. This modification effectively doubles the interaction length without physically extending the device footprint, thereby enhancing phase sensitivity while maintaining compact geometry [3]. A critical advantage of this approach is the improved rejection of common-mode noise: perturbations such as temperature fluctuations or fabrication-induced variations affect both counter-propagating modes symmetrically and are largely canceled out, leading to superior stability of the interference fringes [4]. These features make the LT-MZI particularly attractive for lab-on-chip sensing systems, where device miniaturization and resilience against environmental noise are crucial [5,6,7].

Compared to resonant photonic sensors such as ring resonators [8] and racetrack cavities [9], LT-MZIs offer a wider operational bandwidth and lower susceptibility to fabrication tolerances. Resonant sensors typically achieve high sensitivity through strong field confinement and narrow linewidth resonances but at the cost of reduced dynamic range and increased temperature cross-sensitivity [10]. In contrast, the LT-MZI provides a linear spectral response with respect to refractive index changes, allowing for broader and more robust sensing operation [3]. Fiber-based MZI variants, including core-offset, microbending, and photonic crystal fiber MZIs, have also demonstrated high sensitivity [11,12,13], yet their bulky form factors limit their integration into planar photonic platforms. Recent advances further underline the versatility of LT-MZI structures [14,15]. Subwavelength grating (SWG) waveguides have been shown to dramatically increase the overlap between the guided mode and the analyte, thereby boosting light-matter interaction and nearly doubling sensitivity compared to conventional ridge waveguides [16]. This performance enhancement rivals or surpasses many state-of-the-art interferometric and resonant devices while preserving fabrication simplicity and scalability. Taken together, these attributes establish the LT-MZI as a compelling platform for next-generation photonic sensors, capable of addressing demanding applications in biochemical diagnostics, environmental monitoring, and real-time medical testing, where compactness, stability, and high precision are simultaneously required [1].

The silicon nitride (SiN) platform holds significant importance for integrated photonic sensors due to its unique combination of optical, material, and fabrication advantages [17,18]. With its wide transparency window from the visible to the mid-infrared region, SiN enables versatile sensing across a broad range of biological and chemical analytes. Its low propagation losses and high refractive index contrast support compact device designs with enhanced sensitivity, making it ideal for detecting minute changes in the surrounding environment [19]. SiN is also fully compatible with standard CMOS fabrication processes, allowing for scalable, low-cost production and seamless integration with electronic and microfluidic components. This compatibility not only facilitates mass manufacturing but also supports the development of portable, lab-on-chip biosensing devices [20,21,22]. By combining high performance, scalability, and versatility, the silicon nitride platform provides a robust foundation for next-generation integrated photonic sensors in healthcare, environmental monitoring, and industrial applications [23,24,25].

In this work, we present the experimental realization of an asymmetric loop-terminated Mach–Zehnder interferometer (a-LT-MZI) on the SiN platform for RI sensing. The device incorporates a refractive-index-engineered sensing arm with integrated SWG segments to enhance light-matter interaction while maintaining a compact, CMOS-compatible design. The fabricated structures achieved sensitivities of 288.75–301.25 nm/RIU for standard a-LT-MZI devices and up to 518 nm/RIU with SWG enhancement. To the best of our knowledge, this is the first experimental demonstration of a SiN a-LT-MZI with SWG integration, establishing a scalable and robust platform for lab-on-chip photonic sensors in biochemical, medical, and environmental applications.

## 2. Configuration Framework for the a-LT-MZI Sensor

**Figure 1:**
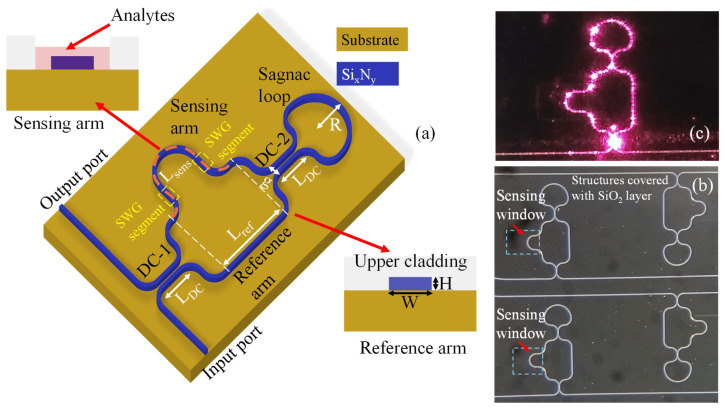
(**a**) Three-dimensional schematic of the LT-MZI structure. For clarity, the upper cladding is omitted in the main illustration. The inset depicts cross-sectional views: the reference arm fully encapsulated with a SiO_2_ upper cladding layer (bottom-right), and the sensing arm featuring an open sensing window (top-left) to enable the introduction of analytes onto the sensing surface. (**b**) Microscope images of the fabricated devices, (**c**) Photograph showing red-light (wavelength = 633 nm) propagation through the sensing structure for demonstration purposes. The devices are designed to operate exclusively in the infrared wavelength range. Note: The SWG segment is incorporated exclusively in the second device configuration, whereas the first configuration employs only a standard ridge waveguide.

In this work, we propose an a-LT-MZI structure on a SiN platform for refractive index (RI) sensing applications. The schematic of the device is illustrated in Figure 1a, and its geometric parameters are summarized in Table 1. The waveguides, with a width of W = 1000 nm and a height of H = 400 nm, are fabricated from SiN, which provides sufficient index contrast with the SiO_2_ cladding to achieve compact waveguide geometries and strong optical confinement. A silicon dioxide (SiO_2_) upper cladding layer is employed to enhance device stability, while a selectively etched sensing window on the sensing arm enables direct interaction between the guided optical mode and the surrounding medium. This interaction modifies the effective refractive index (n_eff_), inducing a spectral shift in the interference pattern, which forms the basis for RI detection. Figure 1b shows a microscopic image of the fabricated a-LT-MZI devices, illustrating four structures with different sensing arm lengths. Figure 1c presents the photograph of an a-LT-MZI structure during demonstration, showing the propagation of red light through the device to highlight its optical behavior and confirm effective light coupling.

In contrast to a conventional MZI, where light propagates unidirectionally through two independent arms, the a-LT-MZI incorporates a Sagnac loop mirror at one end. It should be noted that the rotation-sensitive Sagnac effect is not exploited in this work; the loop functions solely as a reflective element (loop mirror) that folds the propagating waves back into the interferometer. The input light is first split at the directional coupler into the reference and sensing arms. Both beams then propagate toward the loop, where the Sagnac loop mirror reflects them in opposite directions. This reflection causes the two beams to retrace their paths, effectively doubling the optical path length of each arm. The counter-propagating waves return to the coupler, where they recombine and form the output spectrum. Because both waves experience nearly identical environmental perturbations during forward and backward propagation, common-mode noise is largely canceled, while the doubled effective interaction length enhances phase sensitivity without increasing the chip footprint. The resulting interference behavior is governed by the relative phase difference (Δϕ) between the sensing and reference arms. Constructive interference occurs when Δϕ = 2mπ (m ∈ ℤ), producing maximum transmission, whereas destructive interference occurs when Δϕ = (2m + 1)π, leading to minimum transmission. Variations in the ambient refractive index surrounding the sensing arm alter neff, thereby shifting the interference fringes. This mechanism underpins the high precision and sensitivity of the a-LT-MZI in RI sensing applications.

## 3. Optical Characterization

**Figure 2:**
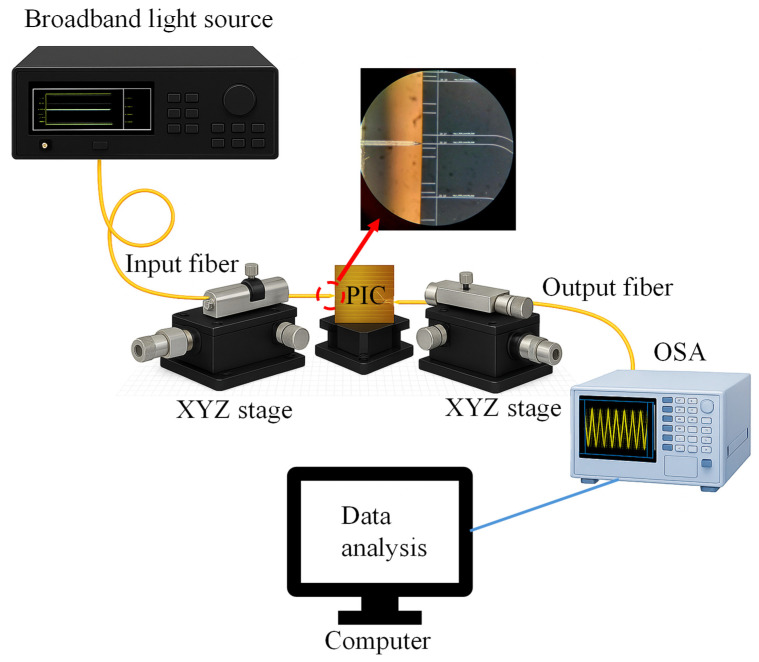
Optical measurement setup for characterizing PICs. Inset: Magnified view of the optical fiber coupled to the PIC.

A broadband light source (DL-BP1-1501A SLED, Ibsen Photonics, Denmark) was coupled into the bus waveguide via a polarization-maintaining tapered fiber, ensuring stable polarization and efficient coupling into the photonic integrated circuit (PIC). The transmitted light was collected with a second tapered fiber and analyzed using an optical spectrum analyzer (OSA, Yokogawa AQ6370B, Japan) to record broadband spectra (Figure 2). To resolve the interference features, the OSA was operated with a 0.02 nm resolution bandwidth and High 2 sensitivity. Certified refractive index calibration liquids (n = 1.408, 1.412, 1.416; Cargille Labs, USA) were applied to the sensing window with a micropipette to ensure uniform coverage of the exposed SiN surface. After each measurement, the chip was rinsed with isopropanol, deionized water, and dried with compressed air to avoid cross-contamination. For precise dip tracking, spectra were also measured with a tunable laser (1520–1620 nm, 0.02 nm step) and fitted with Lorentzian profiles. Four interference dips within 1540–1580 nm were consistently monitored across the RI set, and their linear redshifts confirmed reliable tracking of the interference dips.

**Figure 3:**
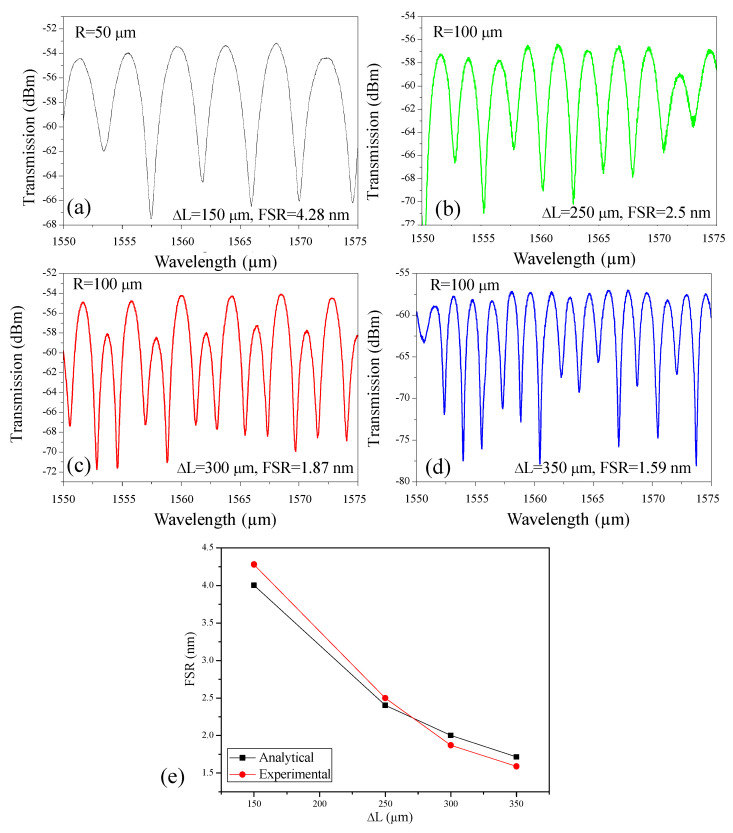
Transmission versus ∆L, (**a**) 150 µm, (**b**) 250 µm, (**c**) 300 µm, (**d**) 350 µm, (**e**) Comparison between analytical and experimental results showing the variation in FSR as a function of ∆L. Note: Spectra are plotted in absolute power (dBm) and were acquired in separate alignments; therefore, absolute levels across panels are not directly comparable.

In the LT-MZI structure, the free spectral range (FSR) represents the wavelength spacing between successive interference fringes in the transmission spectrum. It arises from the optical path length difference (ΔL) between the sensing and reference arms and is mathematically expressed as:

$$FSR=\frac{\lambda^{2}}{2n_{g}\Delta L};$$

where λ is the operating wavelength, and n_g_ is the group index of the waveguide. A larger optical path length difference (ΔL) in a-LT-MZI leads to a stronger phase accumulation between the interferometer arms. This increased phase difference reduces the FSR, resulting in more closely spaced interference fringes across the spectrum. Conversely, when ΔL is smaller, the phase difference grows more slowly with wavelength, producing a wider FSR and more widely separated fringes. This behavior directly reflects the inverse proportionality between FSR and ΔL, and it is one of the most important properties of a-LT-MZIs, as it determines the device’s spectral resolution and effectiveness in filtering and sensing applications (Figure 3).

Figure 3a–d illustrates this effect by presenting the measured transmission spectra of a-LT-MZI structures with arm length differences varying from 150 µm to 350 µm. As ΔL increases, the FSR is observed to decrease systematically from 4.28 nm at 150 µm to 1.59 nm at 350 µm. In an a-LT-MZI configuration, the FSR is determined by the optical path-length difference between the sensing arm and the reference arm. Since both arms terminate in the same loop, the loop radius (R) does not contribute to this difference and therefore does not directly affect the FSR. Instead, the FSR remains governed by the relative lengths of the two arms prior to the loop. Changing the R mainly influences practical aspects of the device, such as footprint and bending losses. A smaller radius (R = 50 µm) results in a more compact device and may exhibit slightly higher bending loss compared to R = 100 µm. Reported measurements indicate that SiN waveguides at 1550 nm maintain low loss for bend radii above ~50–80 µm, with losses dropping below ~0.01 dB per 90° bend for R ≥ 80 µm [26]. Thus, while a 50 µm bend may show marginally higher loss than 100 µm, both radii are within the low-loss regime and are generally acceptable for practical integrated photonic designs.

Moreover, the wavelength dependence of the extinction ratio (ranging from <10 dB to ~20 dB) arises primarily from directional-coupler imbalance, whose splitting ratio varies with wavelength. This effect is further influenced by small propagation-loss differences between the arms and weak facet reflections, which reduce fringe visibility at certain wavelengths. In addition, the full width at half maximum (FWHM) of the interference dips narrows with decreasing FSR, provided that the interferometer finesse remains approximately constant. When the extinction ratio degrades, the finesse is reduced and the dips broaden, partially masking this trend. Figure 3e compares the analytical and experimental results, illustrating the variation in FSR as a function of ΔL. These observations highlight the strong dependence of both spectral spacing and linewidth on path-length imbalance, providing a straightforward means to engineer the spectral characteristics of LT-MZI devices for specific applications.

## 4. Fabrication Procedure of PICs

The photonic integrated circuits (PICs) were fabricated at the Warsaw University of Technology Centre for Advanced Materials and Technologies (WUT-CEZAMAT) using a customized process on 100 mm silicon wafers. The layout was generated with a Python(Version 3.13.7) script employing the Nazca Design Framework. Fabrication began with a sequence of wafer cleaning steps to remove surface contaminants. Standard protocols such as RCA SC1, RCA SC2, and Piranha etching were applied to ensure high surface quality. Subsequently, a wet thermal oxidation step was performed to grow a 2.3 μm silicon dioxide (SiO_2_) layer, which served as the lower cladding and provided electrical insulation from the silicon substrate.

Subsequently, a 400 nm-thick SiN layer was deposited by low-pressure chemical vapor deposition (LPCVD). High-resolution electron beam lithography (EBL) was used to define the device geometry, enabling precise sub-micron patterning. The patterns were etched into the SiN layer using reactive ion etching (RIE), which provided anisotropic profiles with reliable resist selectivity. Although femtosecond laser direct writing is widely used for fabricating fiber gratings and offers flexibility for rapid prototyping, its resolution and material compatibility are insufficient for reliably producing SWGs and high-confinement SiN waveguides [27]. In contrast, RIE provides the nanometer-scale precision, low loss, and CMOS compatibility required for scalable integration of the proposed a-LT-MZI, making it the more appropriate fabrication method.

**Figure 4:**
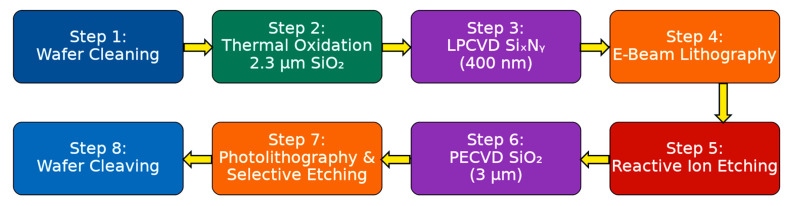
Fabrication workflow of the SiN PIC, illustrating sequential process steps from wafer cleaning to final chip cleaving.

After stripping the resist, a 3 μm-thick SiO_2_ top cladding was deposited by plasma-enhanced chemical vapor deposition (PECVD). To facilitate microfluidic access to the sensing arm, photolithography was employed, followed by selective SiO_2_ etching using a buffered oxide etchant (BOE 7:1, NH_4_F:HF, VLSI-grade purity), which ensured controlled etch depth and high surface quality. Finally, the wafers were cleaved manually into individual PIC chips. The process flow for PIC fabrication is summarized in Figure 4.

**Figure 5:**
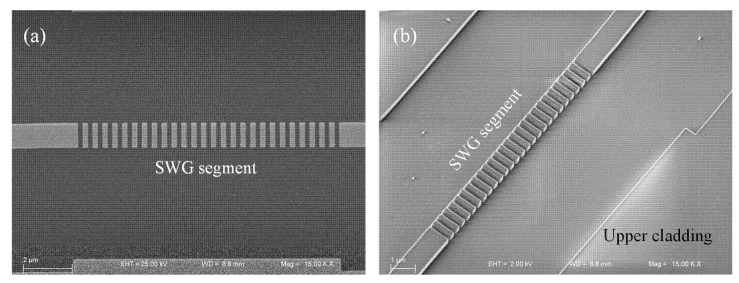
SEM images of the SWG segment integrated into the sensing arm: (**a**) top-view image highlighting the periodic SWG pattern and waveguide geometry, and (**b**) angled-view image providing three-dimensional insight into the structural profile and sidewall quality of the fabricated segment.

Figure 5 illustrates the fabricated SWG segment incorporated into the sensing arm of the a-LT-MZI structure. The top-view scanning electron microscopy (SEM) image clearly reveals the periodic arrangement of the SWG elements and the uniformity of the etched features, confirming high fidelity in pattern transfer (Figure 5a). The angled-view image provides a three-dimensional perspective of the grating profile, highlighting smooth sidewalls and well-defined geometry, both of which are critical for minimizing scattering losses and ensuring reproducible optical performance (Figure 5b). The successful realization of this SWG structure demonstrates the feasibility of integrating engineered gratings within MZI structure to enhance light–matter interaction and tailor device sensitivity.

## 5. Results and Discussion

Refractive index (RI) sensors provide a powerful foundation for the development of advanced biosensors, as they directly measure changes in the optical properties of a medium caused by biomolecular interactions [28]. When a target biomolecule binds to a recognition layer on the sensor surface, the local refractive index shifts, enabling precise and label-free detection. This principle allows biosensors to be designed without the need for additional chemical markers, simplifying fabrication and reducing costs. Moreover, the high sensitivity of RI sensors makes it possible to detect biomolecules at very low concentrations, which is critical for applications such as early disease diagnosis, food safety, and environmental monitoring [29,30,31]. Their compatibility with micro- and nanofabrication also supports the creation of compact, portable biosensing devices. Thus, refractive index sensors serve as the core transduction mechanism that drives the development of efficient, real-time, and miniaturized biosensors for a wide range of biomedical and industrial applications [32].

SWG waveguides have emerged as a powerful platform for enhancing the refractive index sensitivity of integrated photonic sensors due to their engineered light-matter interaction [33]. When the grating period (Λ) is smaller than the guided wavelength (Λ < λ/n_eff_), the waveguide operates in the effective medium regime and can be modeled by homogenization theory. In this work, Λ was set to 400 nm to ensure effective-medium operation in the 1540–1580 nm range while remaining below the Bragg condition and within the fabrication resolution limits. For dielectric SWG waveguides supporting quasi-TE modes, the effective permittivity can be approximated as:

$$\varepsilon_{eff}={FF}_{core}+\left(1-FF\right)\varepsilon_{clad},\;\;\;\;\;\;\;\;n_{eff}=\sqrt{\varepsilon_{eff}}$$

where FF is the duty cycle (fill factor), and ε_core_ and ε_clad_ are the permittivities of the high- and low-index regions, respectively. This tunability allows for precise control over modal confinement and significantly increases the evanescent field overlap with the analyte. The refractive index sensitivity of such a sensor is quantified as:

$$S=\frac{∆\lambda }{∆n};$$

where Δλ denotes the resonance wavelength shift induced by a change Δn in the analyte refractive index [34]. By reducing the average effective index through periodic structuring, SWG waveguides enhance field penetration depth and maximize light-analyte interaction. Consequently, compared to conventional strip or rib waveguides, SWG-based photonic sensors achieve higher sensitivity and figure of merit (FOM = S/FWHM), making them highly suitable for biochemical detection, lab-on-chip diagnostics, and environmental monitoring [35,36].

**Figure 6:**
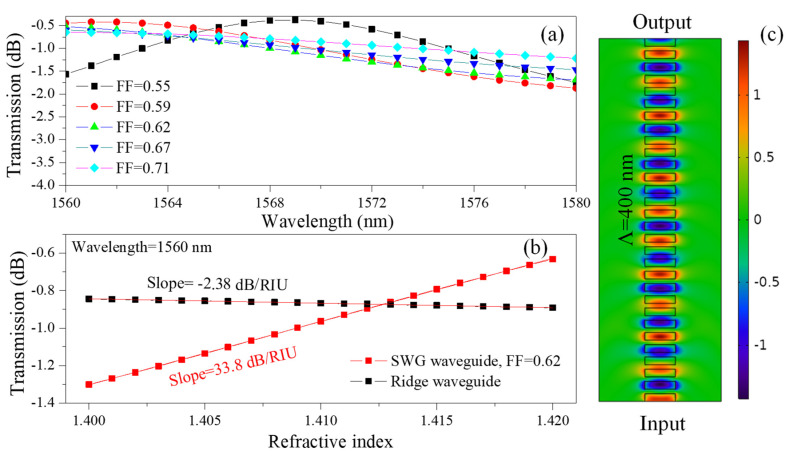
(**a**) Transmission spectra of the SWG waveguide for different FFs within the wavelength range of 1560 nm–1580 nm, (**b**) Transmission response at λ = 1560 nm as a function of refractive index, comparing a standard ridge waveguide and an SWG waveguide (FF = 0.62). The fixed operating wavelength (1560 nm) is marked to clarify the evaluation point for slope extraction, (**c**) Electric field (E_x_) distribution in the SWG waveguide at a wavelength of 1560 nm.

The transmission characteristics of the SWG waveguide were investigated by computing the spectral response within the wavelength range of 1560 nm–1580 nm for different FFs varying between 0.55 and 0.71, as shown in Figure 6a. The propagation loss of an SWG waveguide is influenced by both the FF and the operational wavelength. At low FFs, the optical mode is weakly confined, resulting in significant scattering and radiation losses. Conversely, at very high FFs, the structure begins to resemble a conventional waveguide, where reduced index contrast can also lead to increased loss. An optimal intermediate FF therefore exists, providing a balance between confinement and scattering, and minimizing propagation loss. In addition, the operational wavelength plays a critical role: at shorter wavelengths, the waveguide is more sensitive to fabrication imperfections and surface roughness, leading to higher loss, while at longer wavelengths, scattering loss is reduced, although confinement may be weakened depending on the design. The numerical analysis was carried out using the finite element method (FEM) implemented in COMSOL Multiphysics (Version 6.2), enabling accurate modeling of the electromagnetic field distribution and waveguide transmission behavior.

The transmission spectra of the standard ridge waveguide and the SWG waveguide under varying ambient refractive indices are compared to assess their sensing performance, as shown in Figure 6b. The ridge waveguide exhibits a sensitivity of −2.38 dB/RIU, indicating that transmission decreases with increasing refractive index, whereas the SWG waveguide demonstrates a sensitivity of 33.8 dB/RIU, corresponding to an increase in transmission. Although the sign difference arises from the distinct modal responses of the two structures, the markedly higher magnitude observed in the SWG highlights its superior light-matter interaction and confirms its potential for high-performance photonic sensing applications. Figure 6c depicts the simulated electric field distribution in the SWG waveguide with an FF of 0.62 at 1560 nm, clearly demonstrating the modal confinement and periodic modulation induced by SWG.

### 5.1. Standard a-LT-MZI with Ridge Waveguide

**Figure 7:**
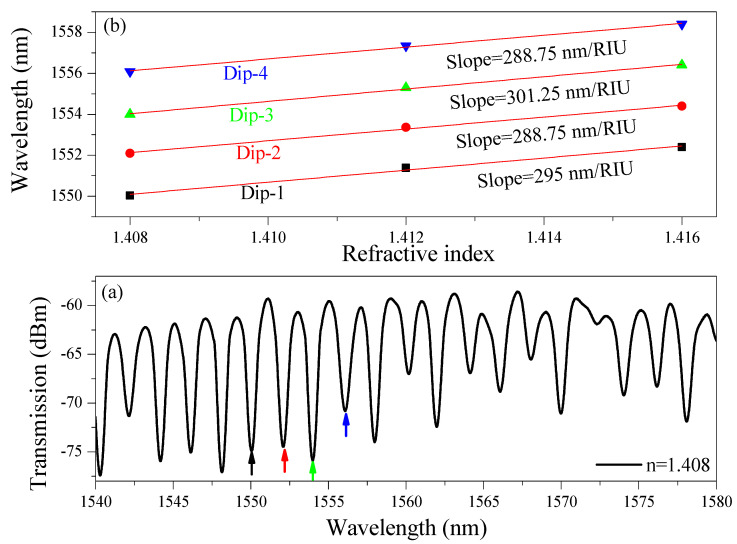
(**a**) Transmission spectrum of the a-LT-MZI structure in the wavelength range of 1540–1580 nm at a surrounding refractive index of 1.408, (**b**) Wavelength shift in the interference dips as a function of refractive index in the range of 1.408–1.416, with experimentally demonstrated sensitivities of 285 nm/RIU (Dip-1), 288.75 nm/RIU (Dip-2), 301.25 nm/RIU (Dip-3), and 288.75 nm/RIU (Dip-4). The consistent redshift in these specific dips across all RI values confirms that the same interference dips were reliably tracked throughout the measurements.

Figure 7a displays the transmission spectrum in the 1540–1580 nm range of the a-LT-MZI when the ambient refractive index is 1.408. Four distinct interference dips (Dip-1 = 1550.04 nm, Dip-2 = 1552.09 nm, Dip-3 = 1553.99 nm, and Dip-4 = 1556.09 nm) are selected in the spectrum, each corresponding to the device’s constructive and destructive interference conditions. The sharpness and regularity of these dips confirm the high-quality interference established in the a-LT-MZI, providing reliable interrogation points for sensing applications. The FWHM of the dips is approximately 0.7 nm. Figure 7b presents the wavelength shifts in the same dips as the refractive index varies from 1.408 to 1.416. A clear and linear redshift is observed for all four dips, indicating that the spectral response of the device is directly and predictably correlated with changes in the ambient refractive index. Experimentally, the sensitivities (nm/RIU) and FOM values were measured as 295 and 421.4 for Dip-1, 288.75 and 412.5 for Dip-2, 301.25 and 430.35 for Dip-3, and 288.75 and 412.5 for Dip-4, respectively. These values fall within a narrow range, showing that the four dips exhibit comparable sensitivity rather than large variations between interference orders. This uniformity is advantageous because it ensures that any of the interference dips may be chosen as the sensing reference without significant compromise in sensitivity. Moreover, the presence of multiple interference dips with similar sensitivities allows for simultaneous interrogation of more than one dip. This approach can improve robustness by reducing the influence of noise, environmental drift, or fabrication tolerances, while also enabling multi-parameter sensing strategies if required.

### 5.2. SWG Segment Integrated a-LT-MZI

**Figure 8:**
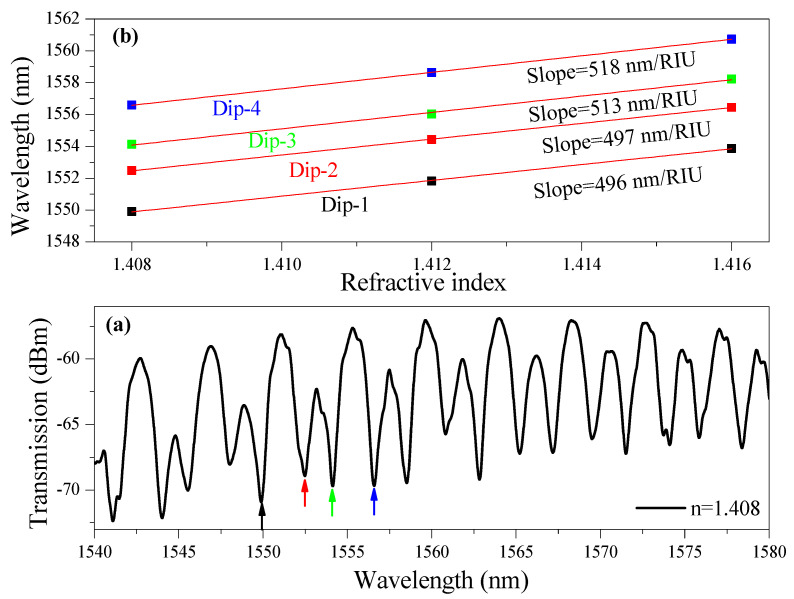
(**a**) Transmission spectrum of the a-LT-MZI structure with an SWG segment in the sensing arm, measured in the wavelength range of 1540–1580 nm at a surrounding refractive index of 1.408. (**b**) Wavelength shift in the interference dips as a function of refractive index in the range of 1.408–1.416, with experimentally demonstrated sensitivities of 496 nm/RIU (Dip-1), 497 nm/RIU (Dip-2), 513 nm/RIU (Dip-3), and 518 nm/RIU (Dip-4). The consistent tracking of the specific dips across all RI values confirms that the same interference dips were reliably monitored in the SWG-integrated device.

To further enhance the sensitivity of the device, a 72-µm-long SWG segment, with 36 µm placed on each side of the bend waveguide, is incorporated into the sensing arm of the a-LT-MZI structure, as illustrated in Figure 1a. Figure 8a illustrates the transmission spectrum of the a-LT-MZI structure incorporating an SWG segment in the sensing arm, measured over the wavelength range of 1540 nm –1580 nm. The interferometer exhibits a path length difference in ΔL = 300 µm, with the sensing arm immersed in a refractive index fluid of n = 1.408. Within this spectral window, four well-defined interference dips, denoted as λ_1_, λ_2_, λ_3_, and λ_4,_ are clearly observed. The FWHM of the dips is approximately 1 nm.

The RI sensitivity of the device was further characterized by applying fluids with refractive indices of n = 1.408, 1.412, and 1.416. The spectral shifts in all four interference dips were monitored, and the corresponding sensitivities are summarized in Figure 8b. The results indicate that the sensitivities and FOM values fall within the ranges of 496–518 nm/RIU and 496–518, respectively, thereby confirming the high sensing performance of the proposed a-LT-MZI configuration.

Finally, Table 2 benchmarks the sensing characteristics of the proposed a-LT-MZI device in comparison with various MZI-based configurations reported in the literature. By covering different material platforms, design architectures, and application domains, the table highlights that the experimentally demonstrated sensitivities of our SiN a-LT-MZI structure are competitive with or superior to many state-of-the-art interferometric sensors.

## 6. Concluding Remarks

We presented the design, fabrication, and experimental demonstration of an asymmetric loop-terminated Mach–Zehnder interferometer (a-LT-MZI) on a silicon nitride platform for refractive index sensing. The use of a Sagnac loop enabled bidirectional light propagation, enhancing phase sensitivity and device stability without increasing footprint. The experimental results showed clear and repeatable interference fringes with linear wavelength shifts in response to refractive index variations. Standard a-LT-MZI devices achieved sensitivities of 288.75–301.25 nm/RIU, while incorporating a 72 µm SWG waveguide segment increased sensitivity to 496–518 nm/RIU. Benchmarking against reported MZI-based sensors confirms the strong performance of the proposed configuration, combining high sensitivity with CMOS compatibility and scalability. These results demonstrate the potential of the a-LT-MZI as a compact and robust platform for lab-on-chip sensing, with applications in biochemical diagnostics, medical testing, and environmental monitoring. Future work will focus on employing alternative waveguide configurations, such as photonic crystal and slot waveguides, in the sensing arm to further enhance device sensitivity and versatility.

## Figures and Tables

**Table 1:** Geometric parameters of the sensor design.

Geometric Parameter	Expression	Value
W	Waveguide height	400 nm
H	Waveguide width	1000 nm
R	Radius of Sagnac loop	50 µm and 100 µm
g	Gap between waveguides	150 nm
L_Dc_	Length of directional coupler	15 µm
L_ref_	Length of reference arm	200 µm to 400 µm
∆L	Difference between the sensing arm and the reference arm (L_ref_−L_sens_)	150 µm–350 µm
Λ	SWG period	400 nm
FF	Fill factor (length of high index segment/grating period)	0.625

**Table 2:** Sensing characteristics of the proposed a-LT-MZI device benchmarked against different MZI structures.

Material Platform	Sensor Configuration	Application	Sensitivity	Numerical/Experimental	Ref.
Silicon nitride	Cascaded MZI	Temperature sensing	710 pm/oC	Experimental	[37]
Silicon nitride	MZI	RI sensing	568 nm/RIU	Numerical	[38]
Silicon-on-insulator	LT-MZI	RI sensing	261 nm/RIU for ridge waveguide; 510 nm/RIU for SWG waveguide	Numerical	[1]
Silicon-on-insulator	MZI with multimode waveguide	RI sensing	826 nm/RIU	Experimental	[39]
Silicon-on-insulator	MZI with SWG slot waveguide	Gas pressure	232 nm/MPa	Numerical	[40]
Silicon-on-insulator	Plasmonic MZI	RI sensing	430 nm/RIU	Numerical	[41]
Silicon-on-insulator	Double slot waveguide-based MZI	RI sensing	700 nm/RIU	Numerical	[42]
Silicon nitride	a-LT-MZI	RI sensing	288.75 nm/RIU–301.25 nm/RIU	Experimental	This study
Silicon nitride	a-LT-MZI with SWG segment	RI sensing	496 nm/RIU to 518 nm/RIU	Experimental	This study

## Data Availability

Data will be available at reasonable requests to the authors.

## Abbreviations

The following abbreviations are used in this manuscript:

a-LT-MZI	Asymmetric loop-terminated Mach-Zehnder Interferometer
PICs	Photonic Integrated Circuits
SWG	Subwavelength grating
RIU	Refractive index unit
FSR	Free spectral range
CMOS	Complementary Metal-Oxide-Semiconductor
FF	Fill factor
